# 2010 ACMP Meeting – Young Investigators' Symposium Abstracts

**DOI:** 10.1120/jacmp.v11i4.3372

**Published:** 2010-07-08

**su‐f‐grandoaksn/o‐1**

## Proton Computed Tomography Reconstruction Using a Bayesian Inference‐based Proton Path Probability Map

D.X. Wang,^a^ T.R. Mackie, W.A. Tome

*University of Wisconsin‐Madison, Madison, Wisconsin*

### Purpose

To describe a method of generating a proton path probability map used for proton computed tomography (pCT) reconstruction.

### Method and Materials

Using the framework of Bayesian inference, a semi‐analytical model based on Fermi's multiple Coulomb scattering (MCS) theory is derived to calculate the conditional probability of a proton passing through each point within an imaging object, given the proton's entrance and exit position and direction. The conditional probabilities at all points form a proton path probability density map (PDM). The most likely path (MLP) is extracted from the PDM, and the PDM is also directly used for image reconstruction. A path ‘envelope’ enclosing 90% of the MLP probability (named MLP90) is extracted for reconstruction to account for the statistical uncertainty. Reconstruction using cubic spline path (CSP) is included for comparison. Data are obtained from Geant4 Monte Carlo simulation.

### Results

The extracted MLP and MLP90 agree well with the real proton path recorded during simulation. Reconstruction using MLP and MLP90 are not significantly different from that using CSP. Reconstruction using the full probability map yields a smoother image than that using MLP or CSP, although it contains artifacts.

### Conclusion

A proton path probability map can be generated using the method described in this abstract. The map can be used directly for pCT reconstruction. MLP and path ‘envelope’ can also be extracted from the map. The reconstructed image using the map, MLP and MLP90 compare well with that using CSP. This method can be used to benchmark different path estimation methods.

**su‐f‐grandoaksn/o‐2**

## Clinical Application of EPID for Routine Machine QA

X. Chen,^1a^ C. Shi,^1^ C. Buckey,^1^ H. Alkhatib,^2^ N. Papanikolaou^2^

*University of Texas Health Science Center,^1^ San Antonio, TX; South Carolina Radiation Oncology Associates,^2^ Columbia, SC*

### Purpose

Increasing interest in electronic portal imaging devices (EPIDs) for radiation dosimetry and other applications such as adaptive radiotherapy has developed over the past decades, particularly with the recent technique advance in high‐resolution amorphous silicon EPID system. For a clinical quality assurance (QA) program, EPIDs may provide a useful QA tool with potential benefits of reduced cost and added convenience such as reduced setup time. This study is to explore the potential clinical application of EPID as a routine machine QA tool.

### Materials and Methods

An integrated EPID‐based QA system was designed, which target to replace conventional machine QA methods. Recommendations by TG‐40 and TG‐45 reports were followed. EPID images were acquired using Varian aS1000 EPID system under integration acquisition mode (6 MV beam; 600 MU/min dose rate). Signal intensity of EPID images were calibrated to dose maps using a linear signal‐dose relationship. Specific EPID‐based QA procedures and image analysis algorithms were designed and developed for some basic QA tasks, such as beam flatness/symmetry test and Winston‐Lutz isocenter test.

### Results

EPID‐measured results were comparable to those measured monthly using films in our institute. For example, EPID‐measured beam quality parameters were: flatness=0.9–1.45%;symmetry=0.48–1.31%;penumbra=3.2–4 mm at SSD=150cm. Star‐shot images were well reconstructed for isocenter check from EPID images acquired at different gantry or collimator angles.

### Conclusion

The results show that EPID may be properly used as a tool for clinical routine machine QA tasks. (Research sponsored by Oncology Data Systems, Inc, Oklahoma City, OK)

**su‐f‐grandoaksn/o‐3**

## Application of 3D Intensity Modulated Brachytherapy for Accelerated Partial Breast Irradiation

B. Guo,^1a^ C. Shi,^1^ C. Cheng,^2^ C. Esquivel,^1^ T. Eng,^1^ N. Papanikolaou^1^

*University of Texas Health Science Center at San Antonio,^1^ San Antonio, Texas; University of Oklahoma Health Science Center,^2^ Oklahoma City, Oklahoma*

### Purpose

To apply 3‐dimensional (3D) intensity‐modulated brachytherapy (IMBT) in accelerated partial breast irradiation (APBI).

### Method and Materials

A dosimetry algorithm for 3D IMBT based on modified TG 43 formulism and Monte Carlo method was proposed and a MATLAB‐based inverse treatment planning system was developed. The system was applied to study the feasibility of 3D IMBT to improve plan quality. Ten intracavitary APBI cases were studied. For each case, an IMBT plan was developed and compared with the original plans used for treatment considering the plan quality, planning and delivery time.

### Results

IMBT plans showed better plan quality compared to the original plans for all cases. For a patient with a small breast and little sparing to ribs and skin, with similar coverage to the target, IMBT plan decreased the high dose region V200 by 16.1%. Maximum doses to skin and ribs were reduced by 56 cGy and 104 cGy in one fraction, respectively. Mean dose to ipsilateral and contralateral breasts and lungs were also slightly reduced by IMBT. The drawback of IMBT is the longer planning and delivery time. IMBT plans take around 2 hours to optimize; the delivery time of IMBT is typical 4–6 times of an isotropic plan.

### Conclusion

Inducing source intensity modulation improves the plan quality of APBI brachytherapy treatment plans, increasing dose uniformity in target and reducing the dose to critical structures. Faster computers and higher output of the source could be solutions to compensate for the increase of plan optimization and delivery time.

**su‐f‐grandoaksn/o‐4**

## Development of the Preliminary Quality Assurance Software for GafChromic EBT2 Film Dosimetry

J. Park,^1a^ J. Lee,^2^ K. Choi,^3^ W. Jung,^1^ T. Suh^1^

*The Catholic University of Korea,^1^ Seoul, KR; Konkuk University Medical Center,^2^ Seoul, KR; SAM Anyang Medical Center,^3^ Anyang, KR*

### Purpose

The software for GafChromic EBT2 film dosimetry was developed. It provides film calibration functions based on each color channel, and evaluations are available about the effects according to the correction methods for light scattering of flat‐bed scanner and thickness differences of active layer.

### Method and Materials

The developed software using MATLAB is composed of two primary frames with multi‐tabs which display imported images and resultant images at each processed step. Dose verification using EBT2 films is implemented following procedures: file import, noise filtering, background and active layer correction, dose calculation and evaluation. Relative and absolute background correction is selectively applied. Calibration results and the fitting equation of sensitometric curve are exported to files. After two dose matrixes from different types of measurement devices or treatment planning system (RTP) are aligned, profiles and isodose curves are compared. In addition, gamma index and gamma histogram are analyzed according to the determined criteria of distance–to–agreement and dose difference.

### Results

Dosimetric results from EBT2 films could be compared with those from RTP, ECLIPSE or 2‐dimensional ionization chamber array, MatriXX. As the performance evaluation was achieved by dose verification in the 60° enhanced dynamic wedged field, absolute dose discrepancy between EBT2 film and ECLIPSE was satisfied within 3% error. In analysis using gamma index applied criteria of 3 mm 3%, 99% pass ratio represented, except for the field edges.

### Conclusion

The software would contribute to recommendation of optimal procedures and the practical QA using EBT2 films.

**su‐f‐grandoaksn/o‐5**

## Independent Dose Calculation Software Development and Validation for Helical Tomotherapy*

W. He,^1a^ L. Vazquez Q,^1^ A. Gutiérrez,^1^ S. Stathakis,^2^ H. Alkhatib,^3^ C. Shi,^1^ N. Papanikolaou^1^

*University of Texas Health Science Center at San Antonio,^1^ San Antonio, TX; CTRC at UTHSCSA,^2^ San Antonio, TX; South Carolina Radiation Oncology Associates,^3^ Columbia, SC*

### Purpose

A second check software platform, *MU‐Tomo*, has been developed to execute independent point dose calculation within seconds for helical tomotherapy modality.

### Method and Materials

*MU‐Tomo* has been designed to verify the planned dose independently. Input parameters required for the software are: archived tomotherapy patient files, initial image coordinates, tomotherapy‐calculated point dose, and machine‐specific dosimetric parameters such as the off‐axis ratios (OARx and OARy), tissue phantom ratios (TPR), and output functions (Scp). The software was validated on four phantom models and 50 tomotherapy patient IMRT plans. The four different phantom studies were: (1) a phantom composed of a 20.0 cm thick rectangular slab of Virtual Water, (2) a step valley phantom in a similar, previously‐mentioned Virtual Water phantom, (3) a monthly output check procedure in a cylindrical cheese phantom (radius 15 cm and length 18 cm), and (4) seven patient quality assurance (DQA) treatment plans in the cheese phantom. Patient plans were selected from various cancer treatment sites.

### Results

Validations of dose differences for four phantom studies were all within 3%. For the 50 patients, examinations show that point dose differences between TomoTherapy TPS and *MU‐Tomo* were within 5.0% for all cases evaluated; 49 of 50 were within 3.3%.

### Conclusion

*MU‐Tomo* can perform an independent dose calculation accurately and quickly, and provide a secondary point dose validation of helical tomotherapy plans.

**su‐f‐grandoaksn/o‐6**

## Experimental and Monte Carlo Estimation of Dose Perturbation Around a Non‐radioactive Brachytherapy Seed due to 6 and 18 MV Photons

J. Steinman,^a^ H. Malhotra

*Roswell Park Cancer Institute, Buffalo, NY*

### Purpose

External beam treatment for patients who have previously had implanted seeds is not standard procedure but an option reserved in salvaging failed prostate brachytherapy treatment. Though small in size, the seeds are many and consist of high‐Z metals capable of causing significant dose perturbation in their immediate vicinity.

### Methods and Materials

Measurements were done with Kodak XV2 and Gafchromic EBT2 films layered above and below a nonradioactive I‐125 seed placed in a grooved Lucite plate. Five cm buildup and 10 cm backscatter were added at 95 cm SSD. The phantom was irradiated with and without seed for $1\times 1{\text{cm}}^{2}$ field with 6 MV photons. DOSXYZnrc simulations were done utilizing same parameters for 1 billion histories. Other measurements using XV films were done comparing the dose perturbation with 18 MV photons and varying depth. Study was further extended to include other nonradioactive seeds (Pd‐103 and Cs‐131), and other metals of various Z. Films were analyzed obtaining cross‐axial profiles of seed and no‐seed determining percent dose perturbation.

### Results

DOSXYZnrc and EBT2 film verified maximum dose enhancement of +15% upstream and −20% downstream of the I‐125 seed surface. XV film showed +6% and −11%. Effect was observed up to ~2 mm upstream and ~5 mm downstream from seed. Dose perturbation was found to decrease with increasing energy but increase with Z and depth of seed in tissue.

### Conclusion

The perturbation of the seeds is highly localized and, as with other heterogeneities, depends on factors such as energy, depth, and material. Future work seeks to evaluate clinical impact of this effect.

**su‐f‐grandoaksn/o‐7**

## Preliminary Characterization of Optically Stimulated Luminescence Point Dosimeters for Diagnostic Energy Use

R. Al‐Senan,^a^ N. Ruiz, M. Hatab

*University of Texas Health Science Center at San Antonio, San Antonio, Texas*

### Purpose

To evaluate the angular dependence and linearity of OSL dosimeters in diagnostic X‐ray energy ranges.

### Method

A diagnostic X‐ray unit was used to evaluate InLight/OSL nanoDot dosimeters. Eight angles were chosen (0°, 45°, 90°, 135°, 180°, 225°, 270°, 315°) reflecting the position of an OSL dosimeter with respect to the X‐ray table. The angular dependence was investigated at four techniques: 81 kVp at 50 mAs and 200 mAs, and 117 kVp at 50 and 200 mAs. Each dosimeter was irradiated at 100 cm SID, centered on a 15×15 cm collimation field and positioned in one of the eight angles. For the linearity, two kVps were tested with a range of mAs from 32 to 400. The stability of the tube output was monitored with a calibrated detector.

### Results

Considering the 0° dosimeter reading as the reference, preliminary results showed a significant reduction in dose at 81 kVp, more pronounced with 50 mAs. A decrease of about 50% was shown in the 90° and slightly less with 270°. Considerable reduction was also observed at 45°, 135°, and 225°. Results for 117 kVp will also be presented. Tube output was highly reproducible throughout the experiment (coefficient of variation: < 0.1%). Linearity results at both kVps showed good linearity (R > 0.99).

### Conclusion

OSL nanoDot dosimeters exhibited excellent linearity but showed a considerable angular dependence in the diagnostic kVp energy ranges used. This could be of concern when such point dosimeters are used to evaluate skin dose in CT.

**su‐f‐grandoaksn/o‐8**

## Signal‐to‐Noise Ratio in Parallel Imaging: ACR's failure and how to overcome it

F. Goerner,^a^ G. Clarke

*University of Texas Health Science Center at San Antonio, San Antonio, Texas*

### Purpose

To determine the ability of four methods of Signal to Noise Ratio (SNR) measurements to represent SNR in partially parallel imaging (PPI) protocols. SNR measurement methods were selected based on their practicality in a standard MRI evaluation by a medical physicist.

### Materials and Methods

A spherical phantom provided by the manufacturer was used in all of the scans, which were performed using a 3T Siemens MRI system. Images using EPI, FSE and TruFISP sequences were acquired. Image data for each sequence were taken without PPI and then with PPI method GRAPPA and PPI method mSENSE using acceleration factors: R = 2, R = 3 and R = 4. Two sets of images were taken and one set with no signal (RF voltage set to 0). Additionally, all protocols were taken with the phase encode oriented in both directions. Four methods of SNR analysis were investigated: N1) Image subtraction method; N2) No signal image method; N4) SD of background method; ACR) ACR method. Image results were analyzed to determine the average g‐factor for each method of SNR calculation and PPI reconstruction method.

### Results

The only method that followed theory in maintaining a g‐factor of greater than one in each one of our protocols was method N1 (image subtraction method). The average g‐factors were as follows (mSENSE/GRAPPA) N1:4.85/4, N2:0.63/‐0.36, N4:0.88/0.09, ACR:0.42/‐0.1.

### CONCLUSIONS

The current method used in SNR evaluation by the ACR does not produce results consistent with theory in the investigated PPI protocols. It is recommended when evaluating PPI protocols that the image subtraction method be used.

